# Morpho-Physicochemical, Nutritional Composition and Phenolic Compound Profile of Two Avocado Landraces in Different Ripening Stages

**DOI:** 10.3390/plants14040624

**Published:** 2025-02-19

**Authors:** Rosa L. Zapata-Luna, Neith Pacheco, Emanuel Herrera-Pool, Angélica Román-Guerrero, Teresa Ayora-Talavera, Soledad C. Pech-Cohuo, Alberto Santillán-Fernández, Juan C. Cuevas-Bernardino

**Affiliations:** 1 Centro de Investigación y Asistencia en Tecnología y Diseño del Estado de Jalisco, A.C., Subsede Sureste, Parque Científico Tecnológico de Yucatán, km 5.5 Sierra Papacal-Chuburná Puerto, Mérida 97302, Yucatán, Mexico; rozapata_al@ciatej.edu.mx (R.L.Z.-L.); npacheco@ciatej.mx (N.P.); ivherrera_al@ciatej.edu.mx (E.H.-P.); tayora@ciatej.mx (T.A.-T.); 2 Departamento de Biotecnología, Universidad Autónoma Metropolitana-Iztapalapa, San Rafael Atlixco, No. 186, Col. Vicentina, Ciudad de México 09340, Mexico; aroman@izt.uam.mx; 3 Departamento de Ingeniería en Robótica Computacional, Universidad Politécnica de Yucatán, Tablaje Catastral 4448, Carretera Mérida-Tetiz Km.4.5, Ucú 97357, Yucatán, Mexico; soledad.pech@upy.edu.mx; 4 SECIHTI—Colegio de Postgraduados Campus Campeche, km 17.5 Haltunchén-Edzná, Sihochac, Champotón 24450, Campeche, Mexico; santillan.alberto@colpos.mx; 5 SECIHTI—Centro de Investigación y Asistencia en Tecnología y Diseño del Estado de Jalisco, A.C., Subsede Sureste, Parque Científico Tecnológico de Yucatán, km 5.5 Sierra Papacal-Chuburná Puerto, Mérida 97302, Yucatán, Mexico

**Keywords:** fruit hardness, bioactive compounds, antioxidant activity, underutilized landraces

## Abstract

Avocado landraces have gained great interest due to their importance in maintaining biodiversity and the presence of bioactive compounds in their fruit, depending on fruit tissues or ripening stages. This study aimed to evaluate the morpho-physicochemical and nutritional components of the peel, pulp, and seed tissues from Lagunero (LA) and Criollo (CA) avocado landraces at different ripening stages. Additionally, phenolic profiles were analyzed by using UPLC-DAD/ESI-MS, and a principal component analysis (PCA) was constructed to determine variations among the determined contents from avocado landraces, fruit tissues, and ripening stages. The CA showed a 30% higher fruit weight and higher percentages of seed (0.52%) and peel (3.62%) weight yields as compared to the LA. Ripening significantly affected the physical characteristics of LA and CA, and a substantial decrease in hardness (83%) after 4 days of storage. In LA, ripening resulted in an increase in fat content in both the peel and pulp. In contrast, CA showed an increase in protein content in the peel and pulp but a decrease in seeds; fat content increased significantly in the pulp of CA and carbohydrates remained the predominant component in all tissues, though they decreased slightly in CA peels during ripening. The ripe LA peel presented approximately 50% more total phenolic compounds than other tissues. The unripe CA peel showed a higher antioxidant capacity according to DPPH (3831.97 µMol Eq Trolox/g dw) and ABTS+ (3674.70 µMol Eq Trolox/g dw) assays. The main phenolic compounds identified in the avocado peel were chlorogenic acid, catechin, quercetin-3-O-hexoside, quercetin-3-O-pentoside, coumaric acid, caffeic acid, neochlorogenic acid, ferulic acid, kaempferol-3-O rhamnoside, and quercetin-3-O-rhamnoside. The PCA analysis revealed a strong correlation between chlorogenic acid and caffeic acid with TPC, while catechin was more closely related to antioxidant activity. These findings suggest that peel and seed tissues of avocado landraces, often considered byproducts, are valuable sources of bioactive compounds with high antioxidant potential.

## 1. Introduction

*Persea americana* Mill., commonly known as avocado, belongs to the Lauraceae family and is extensively grown in tropical and subtropical regions. While its origins can be traced back to Mexico, Central America, or South America, it was initially cultivated in Mexico around 500 BC [1]. The avocado is a fruit with multiple variations, comprising three botanical varieties: *P. americana* var. Americana, *P. americana* var. Guatemalensis, and *P. americana* var. Drymifolia, commonly referred to as the West Indian, Guatemalan, and Mexican varieties, respectively [2]. In Mexico, a country with a rich diversity of avocado genotypes, significant morphological differences have been observed among these varieties, including fruit size, shape, peel texture, and color variations. These differences arise not only from their genetic composition but also from the diverse ecosystems in which they grow, where environmental factors such as climate, soil composition, and altitude play a significant role in influencing the fruit’s size, shape, peel thickness, color, and biochemical composition, including its phytochemical content [3].

The avocado, particularly the Hass variety, is recognized for its rich nutritional profile, including protein, carbohydrates, vitamins, copper, potassium, folate, and monounsaturated fats, as well as significant lipid-soluble compounds that contribute to its health benefits through bioactive components in its lipid fraction [4]. Similarly, native Mexican avocado varieties, although less widely consumed, have been established as functional foods and potential sources of nutraceutical ingredients, including lipids and their derivatives, highlighting their untapped potential for health-promoting applications [5].

Previous studies have identified phenolic compounds like chlorogenic acids, epicatechins, and catechins in avocado tissues using liquid chromatography–mass spectrometry analysis [6,7]. However, most research has focused on measuring phenolic compounds in the pulp, leading to growing interest in avocado byproducts, such as the peel and seeds, which are recognized as excellent sources of antioxidants and bioactive compounds [8]. Notably, the majority of this information relates to the Hass variety, while studies focusing on Mexican landraces remain limited [9,10,11,12]. According to Ramos-Aguilar et al. [11], these avocados exhibit significant genetic diversity, unique morphological traits, and distinct biochemical compositions as compared to commercial varieties. Despite their widespread consumption in Mexico, their nutritional and functional properties remain largely unexplored. Their study identified 38 different phytochemical compounds in Creole avocado fruits with a notable presence of flavones, suggesting significant antioxidant potential [11]. This is particularly relevant in their byproducts such as the seeds, which has also been recognized as a valuable source of bioactive compounds [5].

The Mexican state of Yucatán produced approximately 12 thousand tons of avocados in 2021 [13]. Among these landraces, Lagunero avocado (LA) and Criollo avocado (CA) are commonly consumed as side dishes in traditional regional cuisine. However, the biological diversity of these native fruits is declining, rendering them underutilized resources. To our knowledge, no studies have investigated the morpho-physicochemical nutritional composition and the phenolic profile of LA and CA landraces. Therefore, this study aims to evaluate the morpho-physicochemical properties of fresh fruits to determine their ripe and unripe stages after harvest. Additionally, the nutritional composition, total phenolic content (TPC), and antioxidant activity (DPPH and ABTS+ radical assays) in the pulp, peel, and seed of these avocado landraces were analyzed. Furthermore, their phenolic compositions were characterized through UPLC-DAD/ESI-MS analysis. A comprehensive understanding of these attributes could support the development of innovative products for food industries, enhancing their economic value while promoting biodiversity and the conservation of this unique fruit.

## 2. Results and Discussion

### 2.1. Morpho-Physicochemical Characteristics of Avocado Fruits

**Figure 1:**
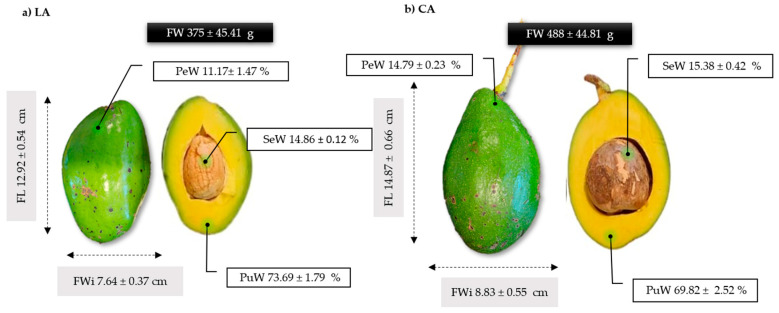
Fruit traits of avocado landrace: (**a**) Lagunero avocado (LA) and (**b**) Criollo avocado (CA). FW: Fruit weight; FL: fruit length; FWi: fruit width; PeW: peel weight %; SeW: seed weight % and PuW: pulp weight %.

The impact of avocado landraces on the fruit’s traits was assessed through the measurement of weight (FW), length (FL), width (FWi), peel weight (PeW), seed weight (SeW), and pulp weight (PuW). The fruit traits of the LA and CA avocados are shown in Figure 1. The fruit trait measurements indicated that the average weight of CA avocados was 30% higher than that of LA avocados. Both LA and CA avocados were considerably heavier as compared to more common commercial varieties like Hass, which has an average fruit weight of approximately 150 g, as reported by Dreher and Davenpor [14]. In this study, the LA avocados were 150% heavier and the CA avocados were 225% heavier than the Hass variety.

Additionally, the CA genotype presented a higher percentage of seed (0.52%) and peel (3.62%) weight yield than the LA genotype. Nevertheless, the LA genotype exhibited a pulp weight yield 3.87% higher than that of the CA genotype. The observed differences in fruit size, total weight, and tissue weight yield among the genotypes can be attributed to the genetic variations inherent to each cultivar. Our findings are in concordance with those of previous studies, which have indicated that genetic and environmental factors influence the variability observed among different avocado genotypes [15]. Given their greater weight, these fruits provide a higher proportion of edible pulp per unit, enhancing efficiency in industrial processing and direct consumption.

To establish the ripening stages of LA and CA landraces, physicochemical properties such as color change, fruit hardness, pH values, total soluble solids (°Brix), total titratable acidity (TTA), and maturity index were monitored over a 4-day storage period (Table 1 and Table 2). The physicochemical properties of LA exhibited significant changes over the 4 days of analysis. L* (Lightness) presented minimal variations (Day 1: 51.8 ± 2.35; Day 4: 48.57 ± 1.23), showing only a slight 6.3% decrease. a* values decreased in greenness by 31.7% from Day 1 (−9.56 ± 0.86) to Day 4 (−6.53 ± 0.81), while b* values, representing the yellow-blue axis, increased by 15.7% (Day 1: 22.93 ± 1.60; Day 4: 26.53 ± 2.25). The color intensity, measured as chroma, also increased by 10% during the same period. A shift toward warmer hues was observed, with a 5.3% reduction in the hue angle (Day 1: 109.76 ± 3.27; Day 4: 103.96 ± 1.95). The LA hardness dramatically decreased by 83.5% (Day 1: 43.37 ± 3.14; Day 4: 7.16 ± 0.64), indicating advanced softening. Regarding chemical properties, the pH increased by 6.4% (Day 1: 6.44 ± 0.11; Day 4: 6.85 ± 0.02), while TTA dropped significantly by 50% (Day 1: 5.46 ± 0.01; Day 4: 2.73 ± 0.01). The maturity index showed a notable increase of 305.6%, from 0.18 on Day 1 to 0.73 on Day 4. These changes highlight the progressive maturation of the Lagunero avocado, characterized by shifts in color parameters, texture softening, reduced acidity, increased pH, and a higher maturity index by Day 4.

Table 2 shows the physicochemical parameters of CA. Significant changes throughout the analysis period were observed. L* values decreased by 15% from Day 1 (53.27 ± 1.70) to Day 2 (45.23 ± 1.60), indicating darkening. a* values showed no significant change, whereas b* values increased slightly by 11.8% from Day 1 (15.83 ± 1.10) to Day 4 (17.70 ± 1.31). Chroma and hue angles remained stable throughout all the days, suggesting no substantial shift in color tone. In terms of texture, the hardness showed a significant reduction of 82.4% from Day 1 (45.28 ± 4.29) to Day 4 (7.96 ± 1.41), indicative of softening during maturation. The pH increased significantly by 20.2%, from 6.05 ± 0.49 on Day 1 to 7.27 ± 0.33 on Day 4, reflecting a decrease in acidity. This was further supported by a 66.6% decrease in TTA, from 8.19 ± 0.02 to 2.73 ± 0.01.

According with Salameh et al. [16] the reduction in TTA in ripened fruit may be attributed to the consumption of organic acids during respiration as the fruit matures, leading to an increase in pH, and it has been proposed that, throughout storage, fruits use organic acids for metabolic processes, which consequently lowers the TTA levels over time, aligning with the findings of this study. °Brix, indicating sugar content, tripled from Day 1 (1.00 ± 0.00) to Day 4 (3.00 ± 0.00). Finally, the maturity index increased by 808.3%, from 0.12 ± 0.00 on Day 1 to 1.09 ± 0.00 on Day 4, marking the progression of fruit ripening.

Although this study aimed to establish the maturity stages of landraces through the analysis of physicochemical parameters over the 4 days of observation, the statistical differences between the landraces were also analyzed (Table S1). It was found that LA exhibited higher values in all color parameters except for a. However, the CA variety showed higher °Brix and TTA, which contribute to a higher maturity index. This may indicate that CA is at a more advanced stage of physiological development as compared to LA, which can be related to its lower chroma, suggesting greener but less intense tones. Therefore, these results can serve as a reference for future studies that focus on the evaluation of the maturity stages between landraces.

Méndez-Zúñiga et al. [17] report that Hass avocados undergo softening and a decrease in firmness as they reach the ripening stage, suggesting optimal firmness values for consumption between 70 and 80 N. However, in our study, the firmness of LA and CA avocados was significantly lower, aligning more closely with other varieties such as the avocados studied by Huaman-Alvino et al. [12], for which the ready-to-eat (RTE) stage is defined within a firmness range of 4–8 N. For instance, Tochihuitl-Martiñón et al. [18] evaluated the “Lonjas” avocado variety and reported a firmness value of 0.32 N at ripeness, which is considerably lower than our findings. Similarly, Ramos-Aguilar [11] observed firmness values ranging from 1.32 to 2.68 N in Creole avocados, which were also considered ripe at these levels. These differences underscore that firmness at the RTE stage is highly dependent on the specific avocado variety being analyzed.

Softening is the primary aspect of the ripening process in avocado fruits, driven by modifications in the composition and structure of the cell wall. According to Marquez et al. [19], these changes are primarily attributed to the hydrolysis of pectic compounds in the cell wall, catalyzed by enzymes such as pectinases, polygalacturonases, cellulases, and amylases. Fruit softening during ripening is further associated with cell wall disassembly, particularly the breakdown of hemicellulose and pectins [20]. The degradation of pectin plays a crucial role in determining fruit firmness, and when combined with enzymatic activity and the loss of cellular turgidity due to transpiration, it ultimately leads to the characteristic softening of avocado fruits [20].

Therefore, based on these physicochemical changes in both LA and CA, avocados from Day 1 were classified as unripe, while those from Day 4 were classified as ripe, and from now on, these stages will be referred to as unripe and ripe, respectively.

### 2.2. Nutritional Composition of Avocado Fruit Tissues

#### 2.2.1. Peel

The nutritional compositions of the peel, pulp, and seeds of unripe and ripe LA and CA avocados were determined on a dry weight basis and are shown in Table 3. The results of the peel analysis revealed carbohydrates as the predominant component (*p* ≤ 0.05), with a higher content in LA (~49%). Fiber was the second most abundant component, more prevalent in CA (~29%). The high fiber content observed in the peels is particularly relevant, given that the thin, smooth texture of these avocado peels makes them occasionally consumed by some individuals, despite the removal of peels presents a processing challenge. Their consumption may contribute to nutritional benefits, as fiber promotes satiety and supports digestive health. The spongy matrix of dietary fiber facilitates the passage of food through the gastrointestinal tract by enhancing intestinal motility, thereby aiding in the prevention of constipation [21]. Ripening significantly influenced the fat content in LA, which increased by ~28.2% (from 4.89% to 6.27%), while in CA, protein content increased from 6.34% to 8.02%. In contrast, carbohydrate content in CA decreased during ripening from 47.71% to 42.74%. These findings highlight the dynamic changes in the proximate composition of avocado peels during the ripening process and the variation between landraces.

#### 2.2.2. Pulp

Carbohydrates and fats were the predominant components in the pulps of both avocado landraces, with a higher content observed in CA. Araújo et al. [22] reported that avocado pulp is primarily known for its elevated lipid content, ranging from 12% to 24%, and predominantly consisting of unsaturated fatty acids, which make up over 70% of the total lipid content. These findings are significant when comparing them with more commercially recognized varieties like Hass, potentially indicating their revaluation. However, it would be interesting to conduct chromatographic analyses to determine the types and quantities of the major fatty acids present in these landraces. Regarding the effect of the ripening stage, a significant reduction in moisture content was observed in LA, decreasing by 15.8% (from 21.23% to 17.88%). A similar effect was observed in the ash content, which decreased by 17.4% in LA (from 4.61% to 3.81%) and by 14.3% in CA (from 5.25% to 4.50%). Viera et al. [23] reported that the amount of ash is associated with the mineral content in foods, and avocado is a highly valued fruit due to its rich mineral content, particularly the potassium (K), magnesium (Mg), phosphorus (P), and calcium (Ca) found in the pulp, which provides significant health benefits such as helping to lower blood pressure and reduce the risk of cardiovascular diseases. Concerning fat content, this parameter increased during ripening in both avocado landraces, rising from 19.14% to 22.55% in LA and from 16.25% to 23.5% in CA. Protein content increased exclusively in CA, from 8.02% to 12.20% during ripening.

#### 2.2.3. Seed

Finally, in the case of the seeds of both avocado landraces, carbohydrates were the predominant component (*p* ≤ 0.05), accounting for 80% in LA and 75% in CA. These results are in concordance with the report that among all the macromolecules found in avocado seeds, carbohydrates are said to make up a significant portion (65%), and starch makes up 90% of the total carbohydrates in avocado seeds [24]. Salazar-López et al. [25] reported a range of carbohydrates between 42% and 81% on avocado seeds, which aligns with our findings. Similarly for fibers, the reported contents ranged from 1.3% to 55% in the seeds [25], consistent with our observations. Changes during ripening were observed exclusively in CA for moisture and fiber content, with moisture increasing from 6.83% to 8.34% and fiber content rising from 4.49% to 7.20%. In addition, the ash content showed a decrease during ripening, with LA declining from 4.61% to 3.81%, while protein content also declined in both genotypes: from 5.59% to 5.21% in LA and from 6.28% to 4.02% in CA. Thus, the significant nutrient content of avocado seeds supports their potential use as an ingredient in human dietary supplements.

Based on all the previous results, the ripe avocado landraces studied contain potential sources of fiber in the peels, fat in the pulps, and carbohydrates in the seeds.

### 2.3. Phenolic Compounds Identification and Quantification by UPLC-DAD/ESI-MS

**Figure 2:**
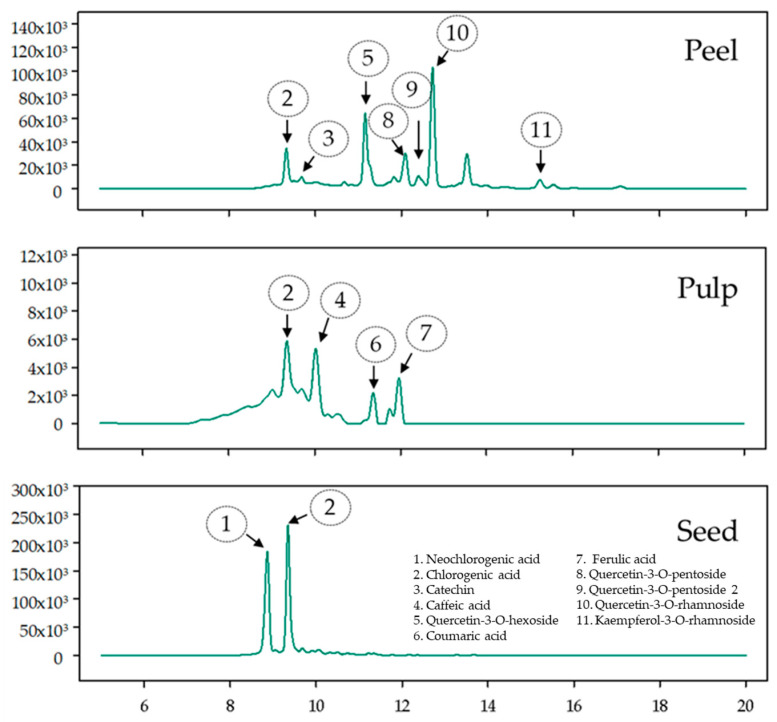
Chromatograms of the main phenolic compounds identified in the peels, pulps, and seeds of avocado landraces obtained by UPLC-DAD/ESI-MS at 350 nm.

The identification of phenolic compounds in the different tissues of LA and CA was performed using the UPLC-DAD/ESI-MS equipment, and quantification was carried out using commercial standards. The 11 main compounds tentatively identified in the evaluated samples (Figure 2 and Figure S1, Table 4) include five hydroxycinnamic phenolic acids (neochlorogenic acid, chlorogenic acid, caffeic acid, coumaric acid, and ferulic acid) and five flavonoids (catechin, Quercetin-3-O-hexoside, Quercetin-3-O-pentoside, Quercetin-3-O-rhamnoside, and Kaempferol 3-O-rhamnoside). The identified compounds, along with their retention times (RT), M-H, and m/z fragments, are presented in Table 4. All identified compounds were detected in the peels of both avocados: seven in the pulps and eight in the seeds of LA and CA. Chlorogenic acid was identified as the predominant compound in both the peels and seeds of LA and CA, with the RCA_Pe_ extracts containing 2.32 µmol Eq chlorogenic acid g^−1^ dw (*p* ≤ 0.05), while the RLA_Se_ and ULA_Se_ extracts contained 1.00 ± 0.06 and 0.78 ± 0.061 µmol Eq chlorogenic acid g^−1^ dw, respectively. These findings align with those reported by Velderrain-Rodríguez et al. [15], who highlighted the derivatives of chlorogenic acid (such as caffeoylquinic acids and coumaroylquinic acids) as the main phenolic compounds in avocado tissues along with other flavonoids, including quercetin glycosides and procyanidins. Chlorogenic acids are known for their significant pharmacological properties, including antioxidant activity, free radical scavenging, and stimulation of the central nervous system [15]. These properties underscore their potential contribution to the TPC observed in avocado peel and antioxidant capacity in seed extracts.

In a recent study conducted by Lyu et al. [7] the researchers identified and quantified the phenolic compounds in Hass, Reed, and Wurtz avocados as found within the pulps, seeds, and peels. Their findings revealed a significant concentration of polyphenols in peels and seeds, aligning with our observations. Notably, among the polyphenol profiles, Reed pulp exhibited higher concentrations of epicatechin, kaempferol, and protocatechuic acid, whereas Wurtz peel contained a higher concentration of hydroxybenzoic acid. In this study, the 11 phenolic compounds tentatively identified were found in the peel, and the highest concentration was chlorogenic acid in RLA_Pe_. Chlorogenic acid (CGA) possesses several notable properties, including anti-inflammatory, anti-hypertensive, and antioxidant effects, and it has potential benefits for lipid and glucose metabolism, particularly in metabolic disorders, and demonstrates hepatoprotective effects by mitigating lipopolysaccharide-induced or chemically-induced liver damage [26]. CGA has also been shown to improve cholesterol profiles by increasing high-density lipoprotein levels and reducing total cholesterol and low-density lipoprotein (LDL) levels, as observed in animal studies [27].

### 2.4. Total Phenolic Content and Antioxidant Activity

**Figure 3:**
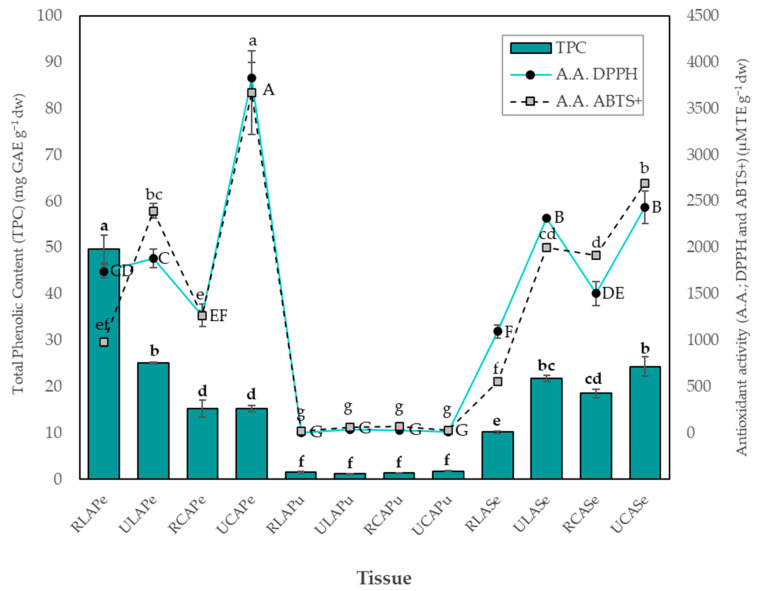
TPC and antioxidant activity against DPPH and ABTS+ radicals of LA and CA tissues. Bars represent the mean ± SD. Different superscript letters (TPC: lowercase bold letter; A.A. DPPH: uppercase letter to the right of the marker; A.A. ABTS: lowercase letter) in the same bar mean significant difference (*p* ≤ 0.05) by the Tukey test. *RLA_P_*_e_: Ripe Lagunero avocado peel; *ULA_Pe_*: unripe Lagunero avocado peel; *RCA_Pe_*: ripe Criollo avocado peel; *UCA_Pe_:* unripe Criollo avocado peel; *RLA_Pu_*: ripe Lagunero avocado pulp; *ULA_Pu_*: unripe Lagunero avocado pulp; *RCA_Pu_*: ripe Criollo avocado pulp; *UCA_Pu_*: unripe Criollo avocado pulp; *RLA_Se_*; ripe Lagunero avocado seed; *ULA_Se_*: unripe Lagunero avocado seed; *RCA_Se_*: ripe Criollo avocado seed; *UCA_Se_*: unripe Criollo avocado seed.

The total phenolic content (TPC) and the antioxidant activity against DPPH and ABTS+ radicals are presented in Figure 3. In the case of TPC, the RLA_Pe_ extract obtained the highest value (49.61 mg GAE g^−1^ dw), followed by ULA_Pe_ (25.13 mg GAE g^−1^ dw) and UCA_Se_ (24.33 mg GAE g^−1^ dw). Comparatively, Gonçalves et al. [28] reported TPC in four regional avocado varieties and one commercial avocado Hass. The TPC of the peels ranged between 11.36 and 20.90 mg GAE g^−1^ dw, and the seeds revealed the highest phenolic content, varying from 3.73 to 24.41 mg GAE g^−1^, which is lower than our findings. In a study by Restrepo-Serna and Cardona-Alzate [29], TPC values of 21.98 and 30.72 mg GAE g^−1^ dw were reported for the peels of Hass and Lorena avocado varieties, respectively, which are lower than the TPC values observed in the peels of the avocado varieties analyzed in this study. This suggests that these avocado landraces could be potential sources of phenolic compounds, which may add value to the fruit and enable its transformation to extract these compounds for applications in the food, cosmetic, and pharmaceutical industries.

Concerning the DPPH and ABTS+ assays, a range of values was observed, from 5.30 ± 0.17 to 3831.97 ± 167.90 µMTE g^−1^ dw for the DPPH radical (Figure 3), and from 16.07 ± 0.26 to 3674.70 ± 496.51 µMTE g^−1^ dw for the ABTS+ radical. The lowest values were exhibited by the pulps of both LA and CA, while the peels represented the highest values, which aligns with the observed TPC. Nevertheless, the extract with the highest antioxidant activity was UCA_Pe_ (DPPH: 3831.97 and ABTS+: 3674.70 µMTE g^−1^ dw) (*p* ≤ 0.05), whereas in the case of the TPC, the highest value was observed in RLA_Pe_, suggesting that the antioxidant activity might be attributed to other components present in the samples. Subsequently, the UCA_Se_ extract showed values of DPPH: 2433.85 and ABTS+: 2695.79 µMTE g^−1^ dw, respectively.

When considering the impact of the ripening stage, a decrease in antioxidant activity was observed in the ripe LA and CA peels and seeds. The reductions were 12.62% (from 1882.54 to 1644.88 µMTE g^−1^ dw) and 66.99% (from 3831.96 to 1265.05 µMTE g^−1^ dw) for the peels of LA and CA, respectively. Similarly, in the seeds, the reductions were 52.76% (from 2317.62 to 1094.94 µMTE g^−1^ dw) for LA and 38.19% (from 2433.85 to 1504.45 µMTE g^−1^ dw) for CA. These findings highlight the significant contributions of the immature stages of both genotypes and components as the most prominent sources of antioxidant activity. According to Uğur et al. [30], plants possess efficient physical and chemical defense mechanisms to combat numerous pathogens and abiotic stressors. Among these mechanisms, the chemical defenses involve compounds, including phenolics, which play a significant role in antioxidant activities, free radical scavenging, and human health. In unripe fruits, the levels of compounds involved in chemical defense mechanisms such as phenolics are notably high.

As observed in the TPC and antioxidant activity peel results, the highest TPC value was found in RLA_Pe_, whereas the antioxidant activity measured by DPPH and ABTS+ was higher in UCA_Pe_. This discrepancy was attributed to factors such as steric hindrance of the extracts’ components during the chemical reaction, which could limit their interactions with DPPH and ABTS+ radicals. Other factors, including polarity, the presence of pigments, or variations in concentration, were also suggested as potential contributors to the underestimation of the extract’s antiradical activity. Furthermore, it was noted that increasing the concentration of certain extract components could negatively impact the performance of the method, leading to underestimated values [31]. Based on these results, a principal component analysis (PCA) was conducted to examine the correlations among the individual concentrations of phenolic compounds, TPC, and antioxidant activity.

### 2.5. Correlation Among TPC, Antioxidant Activity, and Individual Phenolic Compounds

**Figure 4:**
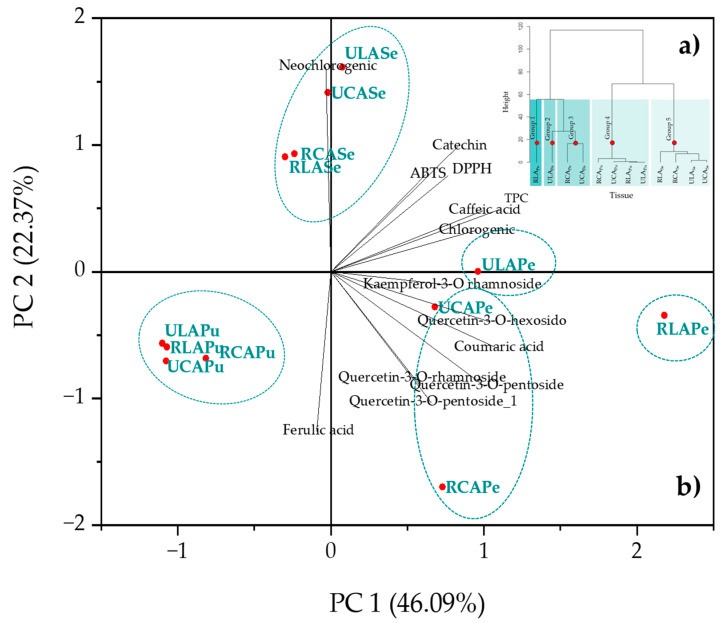
(**a**) Dendrogram and (**b**) principal components analysis (PCA) and the projection of variables including TPC, antioxidant activity (DPPH and ABTS+), and phenolic compounds across different ripening stages and tissues of LA and CA. *RLA_P_*_e_: Ripe Lagunero avocado peel; *ULA_Pe_*: unripe Lagunero avocado peel; *RCA_Pe_*: ripe Criollo avocado peel; *UCA_Pe_:* unripe Criollo avocado peel; *RLA_Pu_*: ripe Lagunero avocado pulp; *ULA_Pu_*: unripe Lagunero avocado pulp; *RCA_Pu_*: ripe Criollo avocado pulp; *UCA_Pu_*: unripe Criollo avocado pulp; *RLA_Se_*; ripe Lagunero avocado seed; *ULA_Se_*: unripe Lagunero avocado seed; *RCA_Se_*: ripe Criollo avocado seed; *UCA_Se_*: unripe Criollo avocado seed.

We conducted a principal components analysis to explore the variations among avocado tissues at different ripening stages. Our analysis utilized TPC, antioxidant activity, and phenolic compound levels as response variables. PCA is a valuable tool that aids in gaining insights into the relationships among samples and variables by reducing data complexity and presenting it graphically (Figure 4). The dendrogram showed five groups (Figure 4a), where PC1 explained 46.09% of the variability and PC2 accounted for 22.37%. Together, these two components explained 68.46% of the total variability. The PCA findings showed that RLA_Pe_ is located separately from the other tissues and is characterized by its high chlorogenic acid content (2.34 µMTE g^−1^ dw) (Table 4), corresponding with its elevated TPC values (Figure 3). However, the weak correlation of RLA_Pe_ with the DPPH and ABTS+ (Table S3) vectors suggests that, despite the abundance of chlorogenic acid, its contribution to antioxidant activity is limited. On the other hand, UCA_Pe_ is strongly correlated with the DPPH and ABTS+ vectors, demonstrating higher antioxidant activity despite lower TPC and chlorogenic acid concentrations (0.14 µMTE g^−1^ dw) (Table 4). This suggests that synergistic interactions among other phenolic compounds, such as catechin, caffeic acid, and quercetin derivatives, may occur in ULA_Pe_ and UCA_Pe_, playing a key role in enhancing radical scavenging capacity (Figure 3).

Statistically, the 31.54% variance not explained by the PCA is due to the presence of diametrically opposed groups with negative correlations, specifically Groups 1, 2, and 3 (peels) in contrast to Group 4 (seed) and Group 5 (pulp). Biologically, this implies that the concentrations of phenolic compounds in Groups 1, 2, and 3 are inversely proportional to their concentrations in Groups 4 and 5. This distribution further supports the idea that the antioxidant potential of the tissues is influenced not only by the total phenolic content but also by the specific composition and interactions of phenolic compounds within each group.

## 3. Materials and Methods

### 3.1. Plant Material

A total of 10 avocado (*Persea americana* var. americana) fruits were randomly selected from a minimum of five trees per landrace (“Lagunero” and “Criollo”). Only healthy fruits, free from pest or disease damage, were chosen. To ensure accuracy in the statistical analysis, the selected fruits were pooled by landrace, and a 10 kg sample was taken for analysis. The fruits were harvested at an unripe stage from a local orchard in Akil, Yucatán, Mexico (20° 14′ 18.0″ N, 89° 19′ 41.8″ W) in July 2021 and were coded as LA and CA, respectively. Immediately, the samples were transported to the Laboratory of Food Safety and Traceability at the Center for Research and Assistance and Technology and Design of the State of Jalisco (CIATEJ) and disinfected with a 0.5% *v/v* sodium hypochlorite solution. Finally, the samples were stored in plastic containers at room temperature (25 °C) until analysis. 

### 3.2. Chemical Reagents

Folin–Ciocalteau reagent, 2,2-diphenyl-1-picrylhydrazyl (DPPH), 2,2′-azino-bis(3-ethylbenzothiazoline-6-sulfonic acid (ABTS+), sodium carbonate (Na_2_CO_3_), potassium persulfate (K_2_S_2_O_8_), (±)-6-hydroxy-2,5,7,8-tetramethylchromane-2-carboxylic acid (Trolox), sulfuric acid (H_2_SO_4_), sodium hydroxide (NaOH), ethyl ether ((CH_3_CH_2_)_2_O), and methanol (MeOH) were purchased from Sigma–Aldrich (Toluca, State of Mexico, Mexico). HPLC acetonitrile (CH_3_CN; ≥99.9%) and formic acid (HCOOH; ≥95.0%) and the analytical standards gallic acid monohydrate (≥98.0%), caffeic acid (≥98.0%), chlorogenic acid (≥95.0%), trans-p-coumaric acid (≥98.0%), ferulic acid (≥98.0%), rutin trihydrate (≥90.0%), (+)-catechin (≥99.0%), and kaempferol (≥90.0%) were purchased from Sigma–Aldrich (St. Louis, MO, USA). Ultrapure water was obtained through a Milli-Q water filtration system (Millipore, Bedford, MA, USA). 

### 3.3. Morpho-Physicochemical Characteristics of Fresh Fruits

#### 3.3.1. Fruit Traits

One day after the fruits were harvested, five fruit samples from each avocado were selected randomly. Then, the length (cm) and width (cm) values were measured using a Vernier caliper (model CALDI-6MP, Truper Co., Ltd., Ciudad de México, Mexico). To quantify the percentage contribution of each fruit component (peel, pulp, and seed), the fruits were cut, separated, and individually weighed to calculate their respective proportions, considering the total fruit weight (FW) of each avocado. All determinations were performed in triplicate.

#### 3.3.2. Color Measurement

The CIE-L*a*b* color parameters of the avocados were conducted in triplicate over 4 days, using a portable MiniScan EZ 4500 L colorimeter (Hunter Associates Laboratory, Inc., Sunset Hills Road, Reston, VA, USA) according to the methodology described by Medina-Torres et al., [32]. Chroma and hue angle values were also analyzed [33].

#### 3.3.3. Hardness

The hardness of each avocado landrace was measured using a universal texture analyzer (model EZ-SX, Shimadzu Co., Ltd., Kyoto, Japan). Twenty fruit samples (five per day over 4 days) were randomly selected and evaluated at unripe and ripe stages using a piercing needle probe (3 mm in diameter) to a depth of 10 mm and at a test speed of 10 cm min^−1^. The maximum force (N) was reported as the firmness of the fruit.

#### 3.3.4. pH Measurements, Total Soluble Solid (°Brix), Total Titratable Acidity (TTA), and Maturity Index

The pH value of each fruit’s pulp was measured with a pH meter (Oaklon, Singapur). Approximately 20 g of the fruit pulp was used to determine the °Brix using a refractometer (Atago PAL-1, Tokyo, Japan) according to the methodology described by Astudiño-Ordoñez and Rodriguez [34]. The TTA content of the avocado pulp was determined using the standard method (NMX-FF-011-1982) and expressed as the percentage of tartaric acid. The maturity index was calculated using the following formula: maturity index = °Brix/TTA [33]. All determinations were performed in triplicate over 4 days. 

### 3.4. Preparation of Samples

The avocadoes’ peel (Pe), seed (Se), and pulp (Pu) tissues from each ripening stage were manually separated using a knife. Then, the yield was obtained for each ratio of the mass (g) of the pulp (MPu), the mass (g) of the peel (MPe), and the mass (g) of the seed (MS) to the total mass (g) of fruit (MF), respectively, per 100. Samples of each avocado landrace were dried using a Freeze Dry System (FreeZone6, Labconco Co., Ltd., Kansas City, MO, USA) at 0.300 mbar and −49 °C for 78 h. The freeze-dried samples were ground using a KitchenAid mill (model BCG111ER, Whirlpool, Co., Ltd., Benton Harbor, MI, USA) until reaching a particle size <500 µm (Mesh ASTM-E11 No.35, RETSCH, Haan, Germany). Finally, the avocado samples were stored at −20 °C until analysis. The codes of each sample are shown in Table 5. 

### 3.5. Nutritional Composition

Moisture, ash, crude fiber, and fat contents were determined using the AOAC standard methods [35]. The nitrogen percentage in the avocado flour was measured by the Kjeldahl method using a VELP system (VELP Scientifica, Usmate Velate, Italy). Then, the crude protein content was calculated using a conversion factor of 6.25. Finally, the total carbohydrate content was calculated by difference. All determinations were performed in triplicate. 

### 3.6. Extraction Procedure of Phenolic Compounds 

The avocado extracts were obtained according to Rozan et al. [36] with some modifications. The phenolic compounds were extracted by an ultrasound-assisted extraction method for each avocado tissue sample (1.0 g) with 20 mL of absolute methanol. Then, testing of the samples was carried out using an Ultrasonic Processor device (model GEX130PB, Sonics and Materials Inc., Newtown, CT, USA) with a diameter probe of 13 mm at an amplitude of 90% for 10 min. A cold bath was used to maintain a temperature below 40 °C. Then, the processed samples were centrifuged using a refrigerated centrifuge (model SL40R, Thermo Fisher Scientific Inc., Dreieich, Germany) at 4500 rpm (4 °C) for 15 min. Subsequently, the supernatants were recovered and filtered using a vacuum filtration system and Whatman 2 filter paper. Then, the samples were collected and adjusted to an initial volume (20 mL) with absolute methanol. Finally, all samples were stored at freezing temperature until analysis. 

### 3.7. Total Phenolic Content (TPC) Determination

TPC was determined according the Žilić et al. [37] methodology with some modifications. Briefly, 100 μL of each extract was mixed with 400 μL of distilled water, 250 μL of Folin–Ciocalteau reagent, and 1250 μL of Na_2_CO_3_ (20% *w/v*). The mixture was vortexed for 5 min and monitored for 40 min at room temperature (25 °C). Absorbance at 725 nm was measured using an UV–vis spectrophotometer (model Biomate 3S, Thermo Fisher Scientific, Inc., Wathan, MA, USA). The TPC content was calculated with the standard curve (50–500 ppm) of gallic acid. Finally, the results were expressed as milligrams of gallic acid equivalents per gram of crude extract sample (mg GAE/g crude extract). All determinations were performed in triplicate. 

### 3.8. DPPH and ABTS+ Radical Assays 

Antioxidant activity by DPPH radical assay was determined according to the procedure of Jimenez-Morales et al. [38] with slight modifications. The extract sample (10 μL) was mixed with 1500 μL of DPPH reagent. Then, the mixture was vortexed vigorously and stored in darkness for 30 min at room temperature (25 °C). The absorbance at 517 nm was measured using an UV–vis spectrophotometer (model Biomate 3S, Thermo Fisher Scientific, Inc., Wathan, MA, USA). Antioxidant activity was obtained with the standard curve from 100 to 700 ppm of Trolox. ABTS+ radical assay was obtained according to the method described by Niang et al. [39]. Briefly, the extract sample (10 μL) was mixed with 1500 μL of ABTS+ reagent. The absorbance was measured at 734 nm. Then, the results of the antioxidant activity were calculated with the standard curve (100–600 ppm) of Trolox. Finally, the results of the DPPH and ABTS+ assays were expressed as micromoles of Trolox equivalents per gram of dry weight sample (µMTE g^−1^ dw sample). All determinations were performed in triplicate. 

### 3.9. Phenolic Compounds Identification by UPLC-DAD-ESI-MS 

The identification and quantification of phenolic compounds were carried out by UPLC-DAD-ESI-MS analysis using an ACQUITY UPLC H-Class system (Waters Co., Ltd., Milford, MA, USA) equipped with a quaternary pump, an automatic injector, and a diode array detector. The separation was carried out using an ACQUITY UPLC BEH C18 (1.7 µm, 2.1 mm × 100 mm) column (Waters, Milford, MA, USA). The mobile phase utilized acidified water (0.1% formic acid in ultra-pure water; solvent A) and acidified acetonitrile (0.1% formic acid in acetonitrile; solvent B). The gradient used was as follows: 0–2 min, 100% A; 2 min, 100–90% A; 2 min, 90–77% A; 1 min, 77% A; 10.5 min, 77–76.5% A; 0.5 min, 0% A; 6 min, 0–50% A; and 6 min, 50–100% A. The flow rate and injection volume were 0.2 mL min^−1^ and 2 µL, respectively. The chromatograms were carried out at a wavelength of 350 nm. The data acquisition and processing for phenolic quantification were performed by Empower 3 software (Empower 3, 2010, Waters Milford, MA, USA). The quantification of phenolic compounds was performed by comparison with the standard curves prepared with the corresponding standard. The results were reported in µM g^−1^ dw. The UPLC analysis was performed in triplicate.

For the mass spectrometry (MS), analysis was conducted utilizing a specific mass spectrometer (Waters XeVo TQ-S micro, MA, USA) as previously described in [38]. The collision energy used ranged from 10 to 150 eV in negative ion mode. Full-scan mode was used to record mass spectra within a range of 50 *m/z* to 700 *m/z*. Data acquisition and processing were performed by MassLynx V4.1 software (Waters, Milford, MA, USA). The tentative identification was determined by comparing their fingerprint and MS data (Figure S1, Supplementary Material) with those documented in the literature, as well as with the public European Mass Bank database.

### 3.10. Statistical Analysis 

The results were expressed as mean ± standard deviation. Data were processed using one-way analysis of variance (ANOVA) and the Tukey means comparison test. Significance was established at *p* ≤ 0.05. To evaluate the effect of the avocado variation (LA and CA), the ripening stages (Unripe and Ripe), and tissues (Pe, Se, and Pu), a principal component analysis (PCA) was used to analyze the effect on the dependent parameters of TPC, antioxidant activity, and phenolic compounds content presented in the samples. Data analysis was conducted using the Statgraphics Centurion XVI software (Statistical Graphics Corp., Manugistics, Inc., Cambridge, MA, USA). 

## 4. Conclusions

This study highlights the influence of landrace and ripening stage on the nutritional compositions, physicochemical properties, and phenolic compound profiles of LA and CA avocados. Ripening led to key modifications in parameters, as reflected in the softening of the fruit, variations in titratable acidity and pH, and increase in the maturity index, particularly evident by Day 4. These changes indicate the progressive ripening of both avocado varieties, impacting their nutritional and functional properties. The total phenolic content (TPC) and antioxidant activity varied across tissues, with notable associations between specific phenolic compounds, such as chlorogenic acid and catechin, as identified through UPLC-DAD/ESI-MS analysis. Peel and seed tissues, often considered byproducts, were found to be valuable sources of bioactive compounds with high antioxidant potential. These findings suggest that LA and CA avocados, especially their underutilized tissues, have promising applications in the pharmacological, food, and cosmetic industries. Moreover, utilizing their bioactive potential could contribute to the conservation and sustainable use of native biodiversity in the Yucatán region.

## Figures and Tables

**Table 1:** Physicochemical parameters of LA fresh fruits over 4 days of analysis.

	Day 1	Day 2	Day 3	Day 4
L*	51.8 ± 2.35 ^a^	53.37 ± 2.35 ^a^	52.93 ± 3.61 ^a^	48.57 ± 1.23 ^a^
a*	−9.56 ± 0.86 ^b^	−8.73 ± 0.64 ^ab^	−7.67 ± 1.11 ^ab^	−6.53 ± 0.81 ^a^
b*	22.93 ± 1.60 ^b^	22.13 ± 1.35 ^b^	26.23 ± 2.32 ^a^	26.53 ± 2.25 ^a^
C* (Chroma)	24.88 ± 1.14 ^b^	23.79 ± 1.51 ^b^	27.33 ± 2.33 ^a^	27.34 ± 2.24 ^a^
(Hue) Angle	1112.74 ± 3.27 ^a^	111.52 ± 2.16 ^ab^	106.24 ± 1.22 ^ab^	103.96 ± 1.95 ^b^
Hardness (N)	43.37 ± 3.14 ^a^	41.86 ± 0.37 ^a^	39.84 ± 2.53 ^a^	7.16 ± 0.64 ^b^
pH	6.44 ± 0.11 ^b^	6.52 ± 0.05 ^ab^	6.715 ± 0.15 ^ab^	6.85 ± 0.02 ^a^
°Brix	1.50 ± 0.72 ^a^	1.50 ± 0.71 ^a^	2.00 ± 0.00 ^a^	2.00 ± 0.00 ^a^
TTA (as% tartaric acid)	5.46 ± 0.01 ^a^	5.44 ± 0.00 ^a^	5.45 ± 0.01 ^a^	2.73 ± 0.01 ^b^
Maturity index	0.18 ± 0.00^c^	0.20 ± 0.02^c^	0.37 ± 0.01 ^b^	0.73 ± 0.00 ^a^

Data Mean ± SD. Different superscript letters in the same row mean significant difference (*p* ≤ 0.05) by the Tukey test.

**Table 2:** Physicochemical parameters of CA fresh fruits over 4 days of analysis.

	Day 1	Day 2	Day 3	Day 4
L*	53.27 ± 1.70 ^a^	41.67 ± 1.71 ^b^	45.83 ± 1.23 ^b^	45.23 ± 1.60 ^b^
a*	−8.5 ± 0.30 ^a^	−8.07 ± 0.67 ^a^	−8.00 ± 0.89 ^a^	−7.73 ± 0.68 ^a^
b*	15.83 ± 1.10 ^a^	16.33 ± 0.95 ^a^	17.33 ± 1.03 ^a^	17.70 ± 1.31 ^a^
C* (Chroma)	17.98 ± 1.37 ^a^	18.22 ± 1.14 ^a^	19.09 ± 1.15 ^a^	19.32 ± 1.43 ^a^
(Hue) Angle	118.30 ± 6.69 ^a^	116.26 ± 0.61 ^a^	114.76 ± 2.12 ^a^	113.60 ± 1.09 ^a^
Hardness (N)	45.28 ± 4.29 ^a^	42.38 ± 1.65 ^a^	40.33 ± 1.02 ^a^	7.69 ± 1.41 ^b^
pH	6.05 ± 0.49 ^b^	6.05± 0.49 ^b^	6.77 ± 0.06 ^b^	7.27 ± 0.33 ^a^
°Brix	1.00 ± 0.00 ^c^	2.00 ± 0.00 ^b^	2.00 ± 0.00 ^b^	3.00 ± 0.00 ^a^
TTA (as% tartaric acid)	8.19 ± 0.02 ^a^	5.44 ± 0.00 ^b^	5.45 ± 0.01 ^b^	2.73 ± 0.01 ^c^
Maturity index	0.12 ± 0.00 ^c^	0.37 ± 0.02 ^b^	0.37 ± 0.01 ^b^	1.09 ± 0.00 ^a^

Data Mean ± SD. Different superscript letters in the same row mean significant difference (*p* ≤ 0.05) by the Tukey test.

**Table 3:** Nutritional composition of different avocado fruit tissues and ripening stages.

Tissue	Moisture (%)	Ash (%)	Fat (%)	Protein (%)	Crude Fiber (%)	Carbohydrate (%)
RLA_Pe_	11.15 ± 0.23 ^c^	3.82 ± 0.74 ^bc^	6.27 ± 0.58^d^	5.96 ± 0.00 ^de^	23.47 ± 0.09 ^b^	48.81 ± 0.26 ^c^
ULA_Pe_	11.03 ± 0.72 ^cd^	4.62 ± 0.62 ^ab^	4.89 ± 0.60 ^ef^	5.96 ± 0.00 ^de^	24.40 ± 0.00 ^b^	49.20 ± 0.98 ^c^
RCA_Pe_	9.98 ± 0.29 ^cde^	3.82 ± 0.06 ^bc^	5.50 ± 0.20 ^de^	8.02 ± 0.27 ^c^	29.88 ± 0.02 ^a^	42.74 ± 0.05 ^e^
UCA_Pe_	9.54 ± 0.17 ^def^	4.42 ± 0.14 ^ab^	3.35 ± 0.14 ^gh^	6.34 ± 0.53 ^d^	28.63 ± 0.89 ^a^	47.71 ± 1.53 ^cd^
RLA_Pu_	17.88 ± 0.22 ^b^	3.81 ± 0.08 ^bc^	22.55 ± 0.01 ^a^	8.58 ± 0.53 ^bc^	10.08 ± 0.01 ^c^	37.10 ± 0.27 ^f^
ULA_Pu_	21.23 ± 0.80 ^a^	4.61 ± 0.13 ^ab^	19.14 ± 0.06 ^b^	9.32 ± 0.00 ^b^	11.16 ± 1.06 ^c^	34.65 ± 2.35 ^f^
RCA_Pu_	7.75 ± 0.70 ^g^	5.25 ± 0.20 ^a^	23.5 ± 0.18 ^a^	8.02 ± 0.01 ^c^	11.64 ± 0.30 ^c^	44.30 ± 0.80 ^de^
UCA_Pu_	8.35 ± 0.91 ^fg^	4.50 ± 0.14 ^ab^	16.25 ± 0.05 ^c^	12.20 ±0.12 ^a^	10.67 ± 0.13 ^c^	48.60 ± 0.19 ^c^
RLA_Se_	1.95 ± 0.10 ^h^	3.20 ± 0.05 ^cde^	4.10 ± 0.13 ^fg^	5.21 ± 0.00 ^e^	5.58 ± 0.36 ^de^	80.03 ± 0.40 ^a^
ULA_Se_	1.84 ± 0.39 ^h^	3.70 ± 0.06 ^bcd^	3.72 ± 0.17 ^gh^	5.59 ± 0.00 ^de^	5.31 ± 0.10 ^de^	80.05 ± 0.37 ^a^
RCA_Se_	8.34 ± 0.38 ^fg^	2.55 ± 0.01 ^e^	2.88 ± 0.37 ^h^	4.02 ± 0.08 ^f^	7.20 ± 0.57 ^d^	75.04 ± 0.49 ^b^
UCA_Se_	6.83 ± 0.22 ^g^	2.77 ± 0.25d ^e^	4.09 ± 0.06 ^fg^	6.28 ± 0.12 ^d^	4.49 ± 0.42 ^e^	75.53 ± 0.51 ^b^

Data Mean ± Standard deviation. Different superscript letters in the same column mean significant difference (*p* ≤ 0.05) by the Tukey test. *RLA_P_*_e_: Ripe Lagunero avocado peel; *ULA_Pe_*: unripe Lagunero avocado peel; *RCA_Pe_*: ripe Criollo avocado peel; *UCA_Pe_:* unripe Criollo avocado peel; *RLA_Pu_*: ripe Lagunero avocado pulp; *ULA_Pu_*: unripe Lagunero avocado pulp; *RCA_Pu_*: ripe Criollo avocado pulp; *UCA_Pu_*: unripe Criollo avocado pulp; *RLA_Se_*; ripe Lagunero avocado seed; *ULA_Se_*: unripe Lagunero avocado seed; *RCA_Se_*: ripe Criollo avocado seed; *UCA_Se_*: unripe Criollo avocado seed.

**Table 4:** Main phenolic compounds identified by UPLC-DAD/ESI-MS in LA and CA extracts.

CN	RT	[M-H]-	(*m/z*)	TI	Concentrations (µM g^−1^ dw)											
					RLA_Pe_	ULA_Pe_	RCA_Pe_	UCA_Pe_	RLA_Pu_	ULA_Pu_	UCA_Pu_	RCA_Pu_	RLA_Se_	ULA_Se_	RCA_Se_	UCA_Se_
1	8.85	353	179, 707, 353, 191	Neochlorogenic acid	0.09 ± 0.00 ^b^	0.04 ± 0.00 ^b^	0.01 ± 0.00 ^b^	NQ	ND	ND	NQ	NQ	0.87 ± 0.02 ^a^	0.83 ± 0.09 ^a^	0.71 ± 0.03 ^a^	0.68 ± 0.03 ^a^
2	9.325	353	707, 353, 191	Chlorogenic acid	2.32 ± 0.05 ^a^	0.83 ± 0.05 ^c^	0.27 ± 0.01 ^de^	0.14 ± 0.01 ^ef^	0.01 ± 0.01 ^f^	NQ	NQ	0.01 ± 0.00 ^f^	1.00 ± 0.06 ^b^	0.78 ± 0.10 ^c^	0.06 ± 0.01 ^f^	0.31 ± 0.02 ^d^
3	9.47	289	245, 289, 205, 203	Catechin	0.55 ± 0.00 ^a^	0.19 ± 0.04 ^bcd^	0.25 ± 0.01 ^bc^	0.13 ± 0.01 ^cde^	0.04 ± 0.01 ^e^	0.02 ± 0.00 ^e^	0.06 ± 0.01 ^e^	0.02 ± 0.00 ^e^	0.24 ± 0.02 ^bc^	0.54 ± 0.07 ^a^	0.28 ± 0.08 ^b^	0.44 ± 0.02 ^a^
4	10.07	179	179, 124	Caffeic acid	0.11 ± 0.01 ^a^	0.05 ± 0.01 ^b^	NQ	0.02 ± 0.00 ^cd^	NQ	NQ	0.01 ± 0.00 ^f^	NQ	0.05 ± 0.00 ^b^	0.04 ± 0.01 ^bc^	NQ	0.02 ± 0.00 ^de^
5	11.151	463	463, 300, 271	Quercetin-3-O-hexoside	0.31 ± 0.06 ^a^	0.16 ± 0.04 ^b^	0.17 ± 0.00 ^b^	0.10 ± 0.00 ^b^	ND	ND	NQ	NQ	NQ	NQ	0.17 ± 0.00 ^b^	NQ
6	11.37	163	119	Coumaric acid	0.12 ± 0.00 ^a^	0.06 ± 0.01 ^b^	0.06 ± 0.01 ^b^	0.06 ± 0.00 ^b^	NQ	NQ	0.03 ± 0.00 ^e^	NQ	0.02 ± 0.00 ^c^	0.01 ± 0.00 ^d^	NQ	0.02 ± 0.00 ^c^
7	11.93	193	--	Ferulic acid	0.04 ± 0.00 ^a^	ND	0.02 ± 0.00 ^c^	ND	0.02 ± 0.00 ^c^	0.02 ± 0.00 ^c^	0.03 ± 0.00 ^ab^	0.03 ± 0.00 ^ab^	NQ	ND	ND	NQ
8	12.1	433	300, 271, 255, 243	Quercetin-3-O-pentoside	0.12 ± 0.02 ^a^	0.06 ± 0.00 ^b^	0.14 ± 0.00 ^a^	0.06 ± 0.00 ^b^	ND	ND	ND	ND	NC	ND	ND	NQ
9	12.36	433	300, 271, 255, 243	Quercetin-3-O-pentoside 2	NQ	NQ	0.05 ± 0.00 ^a^	0.01 ± 0.00 ^b^	ND	ND	ND	ND	ND	ND	ND	ND
10	12.76	447	447, 300, 271, 255, 243, 227	Quercetin-3-O-rhamnoside	0.03 ± 0.00 ^c^	0.02 ± 0.00 ^d^	0.23 ± 0.01 ^a^	0.21 ± 0.00 ^b^	ND	ND	ND	ND	ND	ND	ND	ND
11	15.19	431	284, 255, 227	Kaempferol-3-O-rhamnoside	NQ	0.01 ± 0.00 ^a^	ND	ND	ND	ND	ND	ND	ND	ND	ND	ND

Data Mean ± Standard deviation. Different superscript letters in the same row mean significant difference (*p* ≤ 0.05) by the Tukey test. *RLA_P_*_e_: Ripe Lagunero avocado peel; *ULA_Pe_*: unripe Lagunero avocado peel; *RCA_Pe_*: ripe Criollo avocado peel; *UCA_Pe_:* unripe Criollo avocado peel; *RLA_Pu_*: ripe Lagunero avocado pulp; *ULA_Pu_*: unripe Lagunero avocado pulp; *RCA_Pu_*: ripe Criollo avocado pulp; *UCA_Pu_*: unripe Criollo avocado pulp; *RLA_Se_*; ripe Lagunero avocado seed; *ULA_Se_*: unripe Lagunero avocado seed; *RCA_Se_*: ripe Criollo avocado seed; *UCA_Se_*: unripe Criollo avocado seed. CN: Compound number; RT: Retention time; TI: tentative identification; NQ: not quantified; ND: not detected.

**Table 5:** Avocado landrace tissues at different ripening stages.

Tissue	Avocado Landrace			
	LA		CA	
Pe	ULA_Pe_	RLA_Pe_	UCA_Pe_	RCA_Pe_
Se	ULA_Se_	RLA_Se_	UCA_Se_	RCA_Se_
Pu	ULA_Pu_	RLA_Pu_	UCA_Pu_	RCA_Pu_

*RLA_P_*_e_: Ripe Lagunero avocado peel; *ULA_Pe_*: unripe Lagunero avocado peel; *RCA_Pe_*: ripe Criollo avocado peel; *UCA_Pe_:* unripe Criollo avocado peel; *RLA_Pu_*: ripe Lagunero avocado pulp; *ULA_Pu_*: unripe Lagunero avocado pulp; *RCA_Pu_*: ripe Criollo avocado pulp; *UCA_Pu_*: unripe Criollo avocado pulp; *RLA_Se_*; ripe Lagunero avocado seed; *ULA_Se_*: unripe Lagunero avocado seed; *RCA_Se_*: ripe Criollo avocado seed; *UCA_Se_*: unripe Criollo avocado seed.

## Data Availability

The data presented in this study are available on request from the corresponding author.

## Supplementary Materials

The following supporting information can be downloaded at https://www.mdpi.com/article/10.3390/plants14040624/s1: Table S1. Physicochemical parameters of LA and CA fresh fruits. Table S2. Matrix of percentages and cumulative obtained from PCA of variables TPC, antioxidant activity, and phenolic compounds in LA and CA avocado tissues at different ripening stages. Table S3. Principal components and response variables obtained from PCA of variables TPC, antioxidant activity, and phenolic compounds in LA and CA avocado tissues at different ripening stages. Figure S1. (a) Mass spectra and (b) UV–vis spectrums of tentative identified phenolic compounds in Lagunero and Criollo avocados by UPLC-DAD/ESI-MS. Figure S2. Pearson’s correlation coefficients among TPC, antioxidant activity (DPPH, ABTS+), and individual phenolic compounds from LA and CA.
