# Supplementary material for: Morpho-Physicochemical, Nutritional Composition and Phenolic Compound Profile of Two Avocado Landraces in Different Ripening Stages
Source: Plants (Basel). 2025 Feb 19;14(4):624. doi: 10.3390/plants14040624 (PMC11859218; doi:10.3390/plants14040624)
Supplement: Supplementary file 1 [file plants-14-00624-s001.zip › plants-3461858-supplementary.pdf]

Supplementary Material

Table S1. Physicochemical parameters of LA and CA fresh fruits.

	LA	CA
L*	51.67 ± 2.92[a]	46.50 ± 4.61[b]
a*	-8.13 ± 1.40[a]	-8.08 ± 0.06[a]
b*	24.46 ± 3.03[a]	16.80 ± 1.22[b]
Chroma	25.84 ± 2.77[a]	18.65 ± 1.16[b]
Hue	108.62 ± 4.16[b]	115.73 ± 4.16[a]
Firmness (N)	33.06 ± 15.77[a]	33.92 ± 15.96[a]
pH	6.63 ± 0.18[a]	6.53 ± 0.58[a]
°Brix	1.75 ± 0.40[b]	2.00 ± 0.74[a]
TTA (as % tartaric acid)	4.77 ± 1.23[b]	5.45 ± 2.02[a]
Maturity index	0.37 ± 0.23[b]	0.48 ± 0.38[a]

Means with the same letter per row are not statistically different (Tukey, α = 0.05)

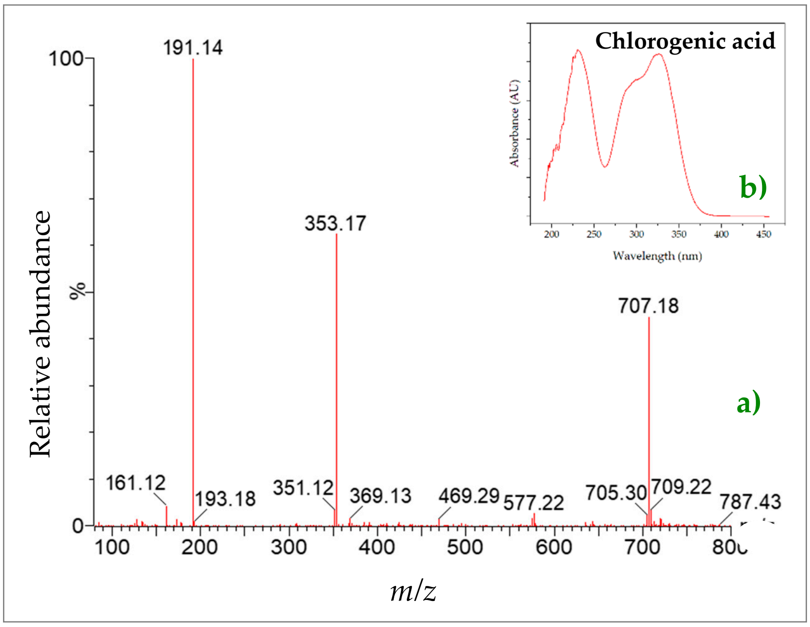

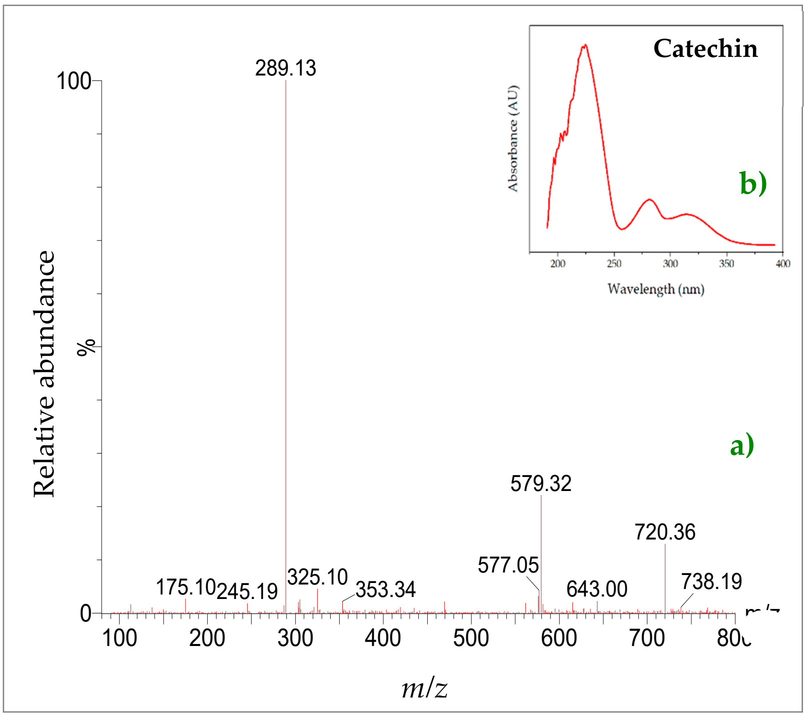
Catechin
Absorbance (AU)
b)
200
250
300
350
400
Wavelength (nm)
289.13
100
Relative abundance
%
a)
579.32
720.36
577.05
175.10
245.19
325.10
353.34
643.00
738.19
0
100
200
300
400
500
600
700
m/z

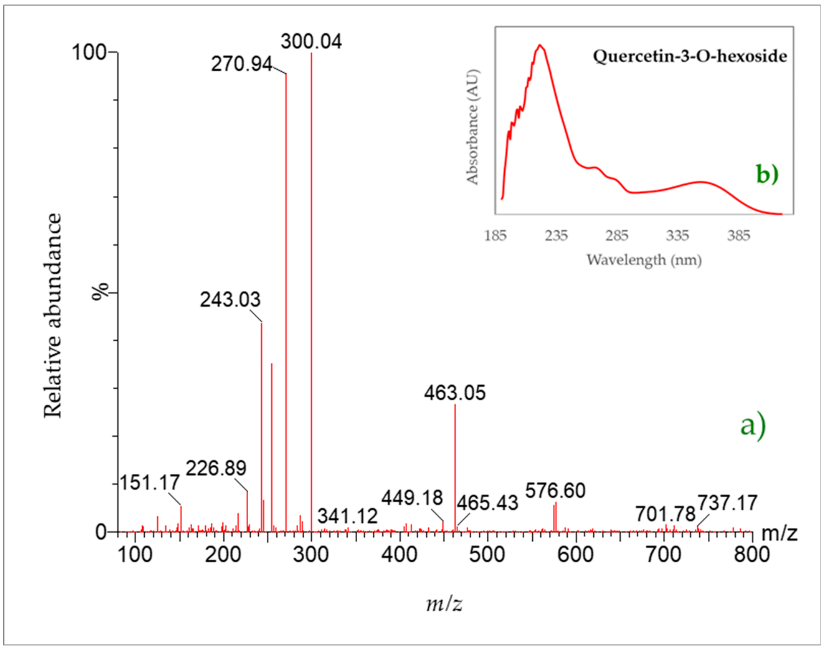
Quercetin-3-O-hexoside
Absorbance (AU)
b)
185
235
285
335
385
Wavelength (nm)
100
300.04
270.94
Relative abundance
%
243.03
463.05
a)
151.17
226.89
449.18
465.43
576.60
341.12
701.78
737.17
0
m/z
100
200
300
400
500
600
700
800
m/z

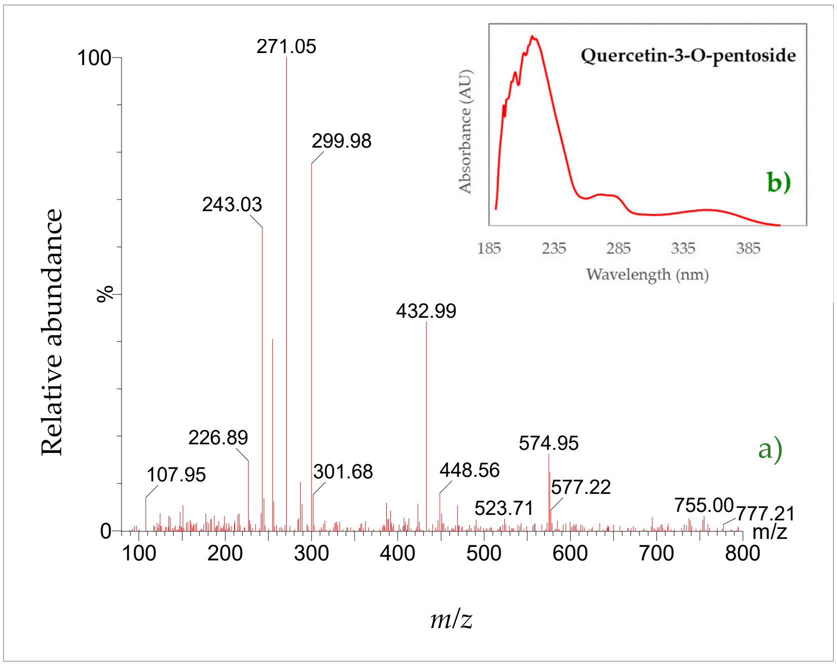
Relative abundance
%
100
0
271.05
299.98
243.03
432.99
226.89
107.95
301.68
448.56
574.95
577.22
523.71
755.00
777.21
m/z
100 200 300 400 500 600 700 800
m/z
a)
Quercetin-3-O-pentoside
Absorbance (AU)
b)
185 235 285 335 385
Wavelength (nm)

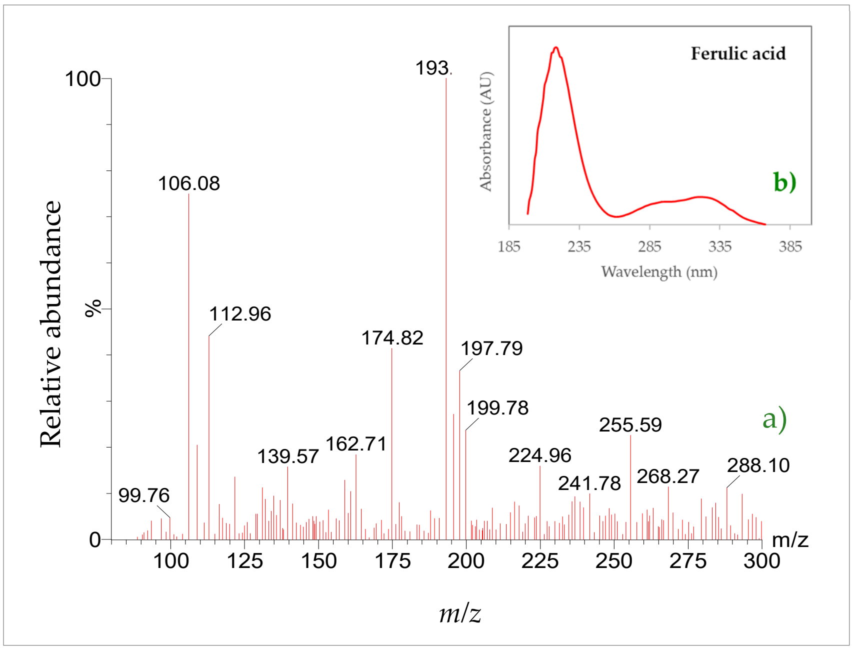
Relative abundance
%
100
0
193.
106.08
112.96
174.82
197.79
199.78
255.59
224.96
241.78
268.27
288.10
139.57
162.71
99.76
m/z
100 125 150 175 200 225 250 275 300
m/z
a)
Ferulic acid
Absorbance (AU)
b)
185 235 285 335 385
Wavelength (nm)

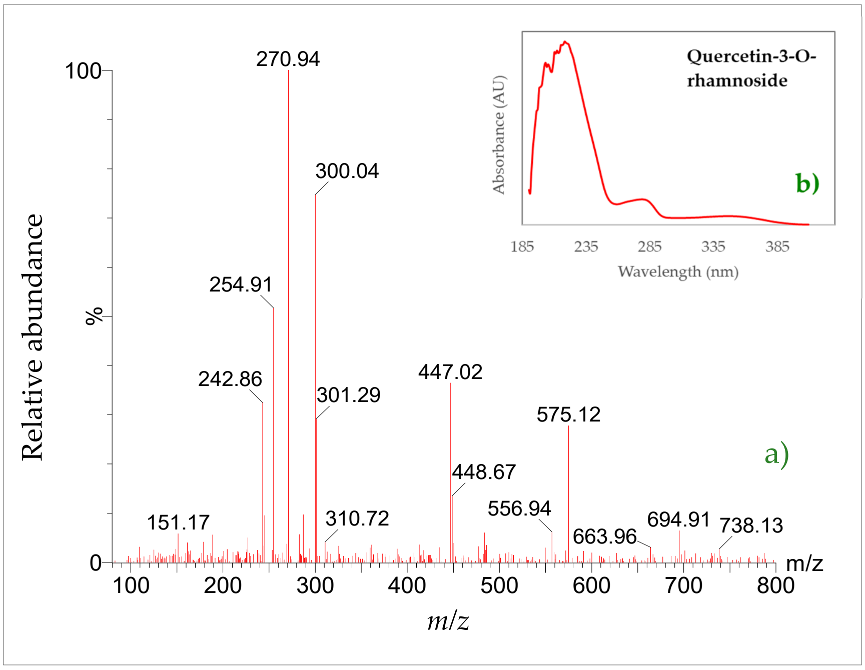

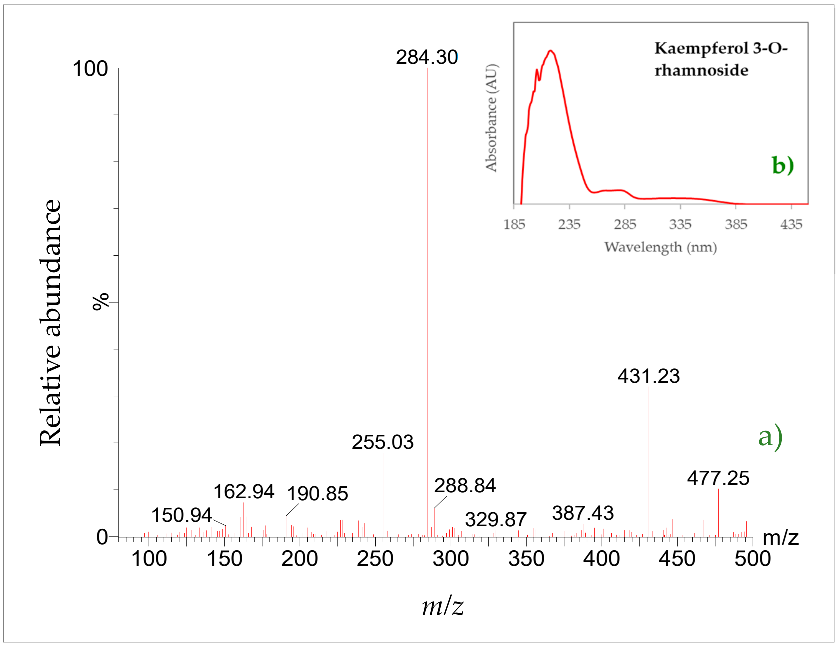

Figure S1. a) Mass spectra and b) UV-vis spectrums of tentative identified phenolic compounds in Lagunero and Criollo avocados by UPLC-DAD-ESI-MS.

Table S2. Matrix of percentages and cumulative obtained from PCA of variables TPC, antioxidant activity, and phenolic compounds in LA and CA avocado tissues at different ripening stages.

PC	Eigenvalue	Percentage of Variance	Cumulative
1	6.45285	46.09%	46.09%
2	3.13129	22.37%	68.46%
3	2.55981	18.28%	86.74%
4	0.88459	6.32%	93.06%
5	0.41153	2.94%	96.00%
6	0.30175	2.16%	98.16%
7	0.1645	1.17%	99.33%
8	0.07645	0.55%	99.88%
9	0.01165	0.08%	99.96%
10	0.00537	0.04%	100.00%
11	2.10E-04	0.00%	100.00%
12	0	0.00%	100.00%

Table S3. Principal components and response variables obtained from PCA of variables TPC, antioxidant activity, and phenolic compounds in LA and CA avocado tissues at different ripening stages.

PC 1	PC 2	
46.09% Loading Plot	22.37% Loading Plot	Loading Plot
-0.01066	0.50694	Neochlorogenic
0.30678	0.10164	Chlorogenic
0.27037	0.31026	Catechin
0.324	0.15116	Caffeic acid
0.33911	-0.12411	Quercetin-3-O-hexosido
0.35467	-0.18966	Coumaric acid
-0.03028	-0.39601	Ferulic acid
0.32771	-0.28401	Quercetin-3-O-pentoside
0.21341	-0.324	Quercetin-3-O-pentoside2
0.19041	-0.28323	Quercetin-3-O-rhamnoside
0.25436	-0.03345	Kaempferol-3-O rhamnoside
0.35916	0.154	TPC
0.24957	0.23799	DPPH
0.20205	0.24091	ABTS

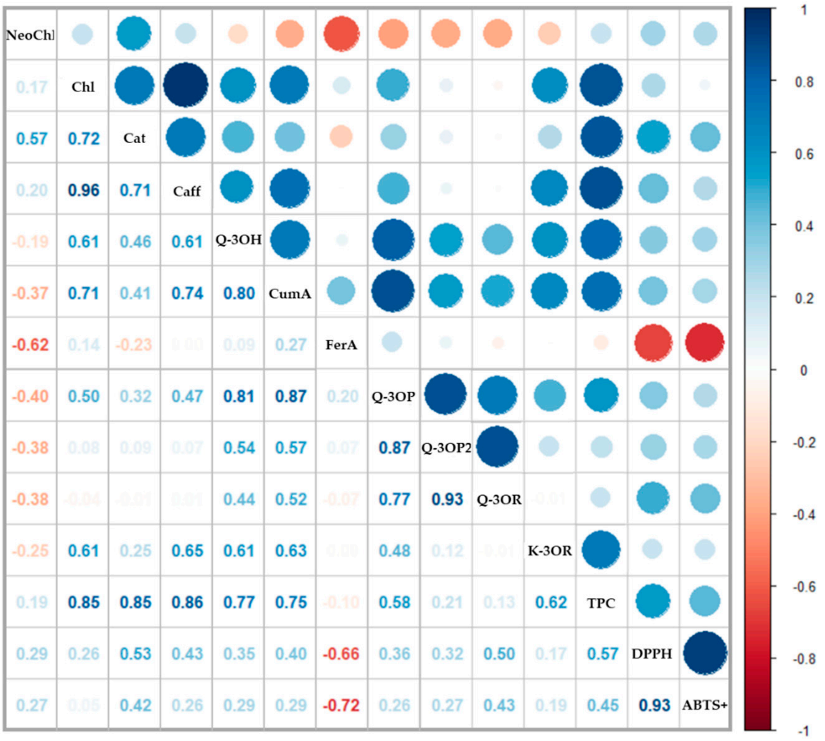

Figure S2. Pearson´s correlation coefficients among TPC, antioxidant activity (DPPH, ABTS+) and individual phenolic compounds from LA and CA. NeoChl: Neochlorogenic acid; Chl: Chlorogenic acid; Cat: Catechin; Caff: Caffeic acid; Q-3OH: Quercetin-3-;O-hexoside; CumA: Coumaric acid; FerA: Ferulic acid; Q-3OP: Quercetin-3-O-pentoside; Q-3OP2: Quercetin-3-O-pentoside 2; Q-3OR: Quercetin-3-O-rhamnoside; K-3OR: Kaempferol-3-O-rhamnoside.
